# An Analytical Study on the Utility of RGB and Multispectral Imagery with Band Selection for Automated Tumor Grading

**DOI:** 10.3390/diagnostics14151625

**Published:** 2024-07-27

**Authors:** Suchithra Kunhoth, Somaya Al-Maadeed

**Affiliations:** Department of Computer Science and Enginering, Qatar University, Doha 2713, Qatar

**Keywords:** multispectral, histopathology, colorectal cancer, infrared image, RGB image

## Abstract

The implementation of tumor grading tasks with image processing and machine learning techniques has progressed immensely over the past several years. Multispectral imaging enabled us to capture the sample as a set of image bands corresponding to different wavelengths in the visible and infrared spectrums. The higher dimensional image data can be well exploited to deliver a range of discriminative features to support the tumor grading application. This paper compares the classification accuracy of RGB and multispectral images, using a case study on colorectal tumor grading with the QU-Al Ahli Dataset (dataset I). Rotation-invariant local phase quantization (LPQ) features with an SVM classifier resulted in 80% accuracy for the RGB images compared to 86% accuracy with the multispectral images in dataset I. However, the higher dimensionality elevates the processing time. We propose a band-selection strategy using mutual information between image bands. This process eliminates redundant bands and increases classification accuracy. The results show that our band-selection method provides better results than normal RGB and multispectral methods. The band-selection algorithm was also tested on another colorectal tumor dataset, the Texas University Dataset (dataset II), to further validate the results. The proposed method demonstrates an accuracy of more than 94% with 10 bands, compared to using the whole set of 16 multispectral bands. Our research emphasizes the advantages of multispectral imaging over the RGB imaging approach and proposes a band-selection method to address the higher computational demands of multispectral imaging.

## 1. Introduction

Cancer has emerged as a threatening disease, with its rising incidence and high mortality rate in recent years. Research has so far yielded treatments that only modestly extend life expectancy. However, early screening can identify the disease at an earlier stage, which, in turn, promotes a better life-saving treatment outcome [1,2]. Histopathological analysis, or biopsy, is regarded as the primary screening method for most cancers. Automating the inspection of biopsy samples allows for screening a larger number of individuals in a shorter period of time while maintaining or even improving diagnostic accuracy. These tools can also be cost-effective [3]. Additionally, digital histopathology can aid in several applications, such as predicting genetic alterations and identifying prognostic biomarkers from biopsy slides [4]. In image-processing-based methods, features are extracted from digitized biopsy samples to differentiate between various cell types or tumor grades. Images of the biopsy samples can be acquired in the form of RGB and multispectral images. Multispectral imaging provides both spectral and spatial information. Moreover, imaging the sample in the infrared bands is also possible with multispectral imagery.

Automated systems to detect and classify cancerous regions are not a novel research area. Several algorithms have been proposed to build such diagnostic systems by utilizing biopsy images. This approach provides faster screening for cancers affecting different body parts, including, but not limited to the breast, cervix, colon, and prostate. The automatic grading of breast cancer cells into three malignancy levels was performed in [5]. Shape and textural features could achieve an accuracy of up to 94.24% with an SVM classifier. The dataset was comprised of 110 biopsy images. Statistical and textural features were utilized in [6] for the grading of breast cancer into three malignancy grades. The system yielded 90% accuracy using a probabilistic neural network, where the images considered were taken under lower magnification. Apart from using hand-crafted features, Refs. [7,8] relied on convolutional neural networks (CNNs) for breast cancer cell classification. The texture analysis method deployed in [9] makes use of a grey-level run length matrix for feature extraction to differentiate prostate cancer cells from normal ones. After analyzing 42 cancerous and 6280 normal regions, the algorithm achieved a classification accuracy of 89.5% with a multilayer perceptron classifier. Haralick’s Grey-Level Co-occurrence Matrix (GLCM) feature-based algorithm was implemented in [10] for the Gleason grading of prostate cancer. A four-class grading scheme delivered an 87% rate of correct grading of the samples. Wavelet and fractal features for prostate cancer detection were investigated in [11,12]. An accuracy close to 97% was claimed in [12] with the usage of an SVM classifier.

A colorectal tumor-classification system was proposed in [13]. The benefits of several feature extraction techniques, such as HOG and color-component-based statistical moments, were exploited by Haralick to build the algorithm. Using an ensemble classifier, the system could detect cancer at a recognition rate of 98.85%. Detecting and classifying cell nuclei in cancerous tissue is a significant task, which has been achieved using a spatially constrained convolutional neural network in [14]. The experiments performed on 20,000 annotated nuclei belonging to four different classes indicated better performance compared to several works to date. The algorithm in [15] for cancer classification from microscopic biopsy images was performed on four types of tissues, such as connective, epithelial, muscular, and nervous. A k-means segmentation algorithm was followed by the extraction of a range of features, including texture, morphology, color, tamuras, and wavelet. An accuracy of 92.19% was reported for the dataset of 2828 images.

A streaming implementation of convolutional layers was employed to train a ResNet CNN for detecting prostate cancer from whole-slide images (WSIs) of biopsies [16]. This approach demonstrated that it could extract meaningful features from high-resolution images and deliver comparable performance to patch-based and multi-instance methods, utilizing whole-slide labels instead of pixel-level annotations. Another deep neural network (DNN) algorithm was proposed in [17] to detect and grade cancer in prostate biopsy samples. This research used two convolutional DNN ensembles: the first ensemble performed binary classification of image patches into benign or malignant and the second ensemble classified the patches into Gleason patterns from 3 to 5. The system achieved an area under the ROC of 0.997 for distinguishing benign from malignant tissue. A transformer-based holistic attention network [18] was introduced to classify breast biopsy images into four categories, benign, atypia, ductal carcinoma in situ, and invasive breast cancer, achieving classification accuracy comparable to 87 U.S. pathologists. A deep learning model based on vision transformers was presented in [19] for Gleason grading in prostate biopsy images. This system extracted patches from the detected regions of interest in WSIs, followed by the application of vision transformers for classification, and achieved a recall rate of 79.8% on 10,616 WSIs. The two-stage transformer-based multiple instance learning method [20] was used for the classification of WSIs of breast cancer pathology. The first-stage hierarchical swin transformer captured global and local information of pathological images, while the second-stage transformer encoder produced powerful bag-level features for classification. Connectivity-aware Graph transformers were used for the classification of breast cancer subtypes in [21]. Although this method surpassed state-of-the-art methods, it was only validated on patch-level image datasets.

The papers discussed so far make use of RGB images of biopsy samples. Multispectral image (MSI)-based tumor-grading systems are also available, with which the rich information from different spectral bands could also be utilized. Raquel et al. [22] proposed a technique for in vivo brain tumor detection using hyperspectral imaging (HSI). Here, data are acquired simultaneously in the spatial dimension and numerous spectral wavelengths. The resultant image comprises the reflectance values, which indicates the fraction of electromagnetic radiation that is reflected from the particular material surface. This capability of HSI enables material identification and, hence, tissue classification. This non-invasive nature of HSI has been exploited in [23,24,25] for the classification of skin cancer into Benign Epithelial, Benign Melanocytic, Malignant Epithelial, and Malignant Melanocytic classes. In addition to these in vivo cancer-detection approaches, HSI/MSI has also been used for the analysis of digitized histopathological specimens. A review of multispectral imaging and machine learning techniques for cancer detection has been given in [26]. This study discusses the works that utilize multispectral images of biopsy samples for cancer cell classification. The image acquisition described in those papers consisted of generating multispectral image cubes from visible bands of the electromagnetic spectrum. Another review [27] explores a broader application of HSI, which covers areas such as staining and color correction, immunohistochemistry, and autofluorescence, in addition to the histopathological analysis. Moreover, it includes a number of major diseases including cancer. In contrast to those experiments, one of our prior works [28] applied multispectral imaging of biopsy samples in the near-infrared range.

Numerous studies in the literature have employed RGB or multispectral imaging modalities for the digitization of histopathological slides. These images have been independently used to develop systems for detecting and classifying various tumor categories. However, a comparison is essential to comprehend the relative advantages and limitations of each modality in automated tumor grading. Our paper offers two main contributions. First, we compare tumor grading approaches using RGB and multispectral images. Second, we applied a band-selection method to reduce the dimensionality of multispectral image data. In this paper, we conduct a comparative study to explore the benefits of multispectral imaging over RGB for colorectal grading applications. Texture feature extraction followed by an SVM classifier is used to differentiate the four major types of tumor cells captured in both RGB and multispectral images. Processing a multispectral image cube is more computationally complex than processing a standard RGB image. Therefore, we propose a band-selection strategy based on the mutual information between image bands to select the most informative bands. We also tested this band-selection approach on another colorectal tumor dataset, which showed a significant improvement in classification accuracy.

The paper is organized as follows. Section 2 gives a brief description of our data acquisition procedure, an overview of the proposed band-selection methodology, and the feature extraction and classification stages. The experimental results for the comparison of the imaging modalities, as well as for the proposed band selection are shown in Section 3. A discussion is provided in Section 4, followed by the Limitations and Future work in Section 5 and the conclusion in Section 6.

## 2. Materials and Methods

This section details the methodology used in our study. We start with a description of the data acquisition process, including the types of colorectal tissue samples utilized and the imaging techniques applied. Next, we elaborate on the band-selection technique employed to improve the performance of our multispectral imaging system, followed by the feature extraction and classification algorithms. Lastly, we discuss the performance metrics used in this research.

### 2.1. Image Acquisition

**Figure 1:**
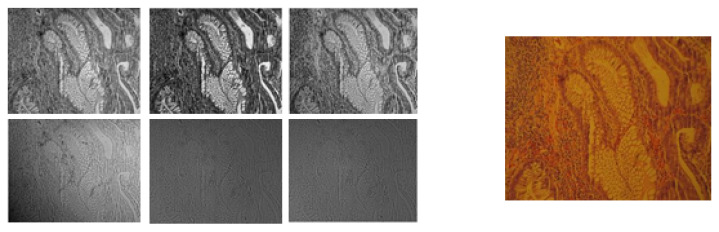
Sample images from ‘tubular adenoma class’: (**Left**) Multispectral image bands (4 VIS bands followed by 2 NIR bands). (**Right**) RGB image.

Our research, which includes the collection and analysis of biopsy samples from Al-Ahli Hospital, received approval from both the Qatar University Institutional Review Board (QU-IRB) and the Al-Ahli Hospital Ethical Committee. The biopsy samples were collected from the Pathology and Laboratory Medicine lab of Al Ahli Hospital, Qatar. The specimens are H&E-stained and comprise normal, hyperplastic, tubular adenoma with low-grade dysplasia, and the carcinoma grades of colorectal cells. We collected a total of 164 colorectal biopsy slides between 2007 and 2016. From these slides, we obtained 200 images, each of which was divided into 4 patches, resulting in 800 individual images for analysis. The biopsies were collected and processed in accordance with standard clinical procedures to ensure the consistency and reliability of the samples. The multispectral image acquisition of the samples is detailed in [28]. We have a 320 × 256 × 39-dimensional multispectral cube corresponding to the 200 samples belonging to the 4 classes (dataset I). The same samples were then acquired using a Canon power shot A650 IS camera to obtain the RGB digitized version. Figure 1 shows examples of the tissues in the multispectral and RGB images. The six sub-images on the left display the multispectral images of a tubular adenoma sample, with the first four bands from the visible spectrum and the next two from the NIR spectrum. The right side shows the same sample captured by an RGB camera.

### 2.2. Band-Selection Method

**Figure 2:**
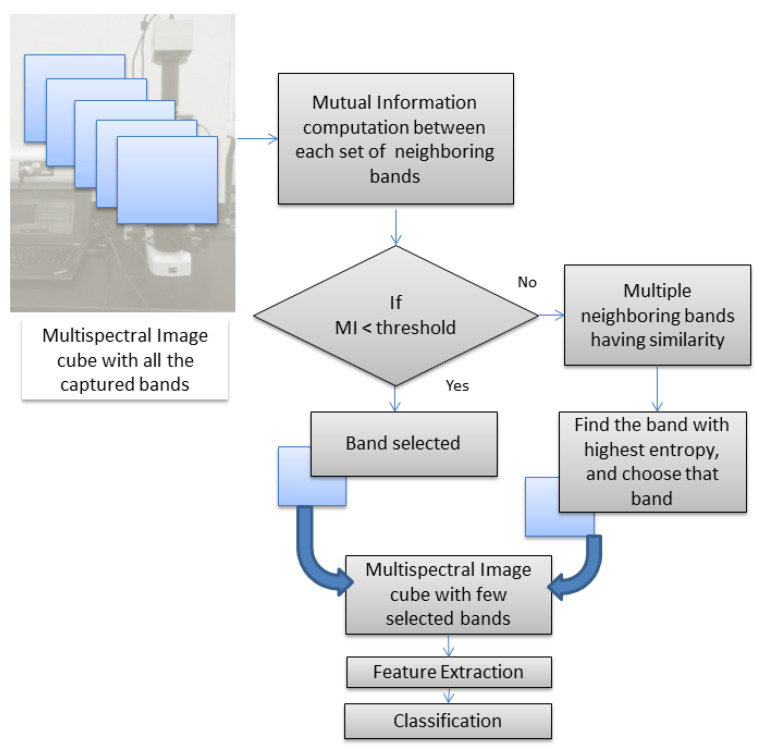
Proposed methodology for band selection.

In this paper, we aim to explore the efficiency of multispectral imaging technology for a computer-aided cancer diagnostic system. Our dataset consists of multispectral images in both the visible and infrared bands and can fully demonstrate the significance of using both type of bands compared to mere RGB data. It is obvious that the numerous bands coming in the multispectral image cube can increase the processing time in proportion to the number of bands included. Apart from the comparative study, we introduce a band-selection approach that can reduce the computational burden of huge data. The methodology is depicted in Figure 2.

With a multispectral image acquisition system, we are able to capture numerous bands of images from a sample. This may include visible and infrared ones. It is expected that different modes of information can be perceived from the multiple bands. However, there is a possibility that too much information can decrease our computational accuracy. The discriminative capability of a classifier deteriorates with redundant data. It is not practical to quantify the range of information contained in each of the bands at the image acquisition stage. Therefore, this process must be effectively managed as a band-selection approach after the initial epoch. This approach could yield a higher classification rate, which demonstrates that certain unwanted outlier bands have been successfully eliminated. A better classification could result in reduced processing time as well. It would be better to capture sufficient bands in the first set of samples from a particular setup. Once the information-rich bands are identified following the band-selection methodology, further acquisition can make use of only the required set of bands.

Many band-selection algorithms have been developed to date for dimensionality reduction. Our proposed approach is based on mutual information. In information theory, mutual information quantifies the statistical dependence between two random variables [29], indicating how much information one variable provides about another. It has been used as a tool for image registration, where it measures the similarity between two images. Mutual information MI(A,B) measures A–B dependence by measuring the distance between a joint distribution $p_{AB}\left(a,b\right)$ and the distribution associated with complete independence, represented as $p_{A}\left(a\right)\cdot p_{B}\left(b\right)$.

(1) $$MI\left(A,B\right)=\sum_{\left(a,b\right)}p_{AB}\left(a,b\right)\log\left(\frac{p_{AB}\left(a,b\right)}{p_{A}\left(a\right)\cdot p_{B}\left(b\right)}\right)$$

The mutual information can also be expressed in terms of the entropy. Entropy is a measure of uncertainty regarding a particular random variable. High entropy images correspond to good-contrast ones and, hence, have abundant information.

(2) $$\begin{array}{cc} MI(A,B) & =H(A)+H(B)-H(A,B) \end{array}$$

(3) $$\begin{array}{cc} \; & =H(A)-H(A|B) \end{array}$$

where

- H(A) is the entropy of random variable *A*;
- H(B) is the entropy of random variable *B*;
- H(A,B) is the joint entropy of *A* and *B*;
- H(A|B) is the conditional entropy of *A* given *B*.

**Figure 3:**
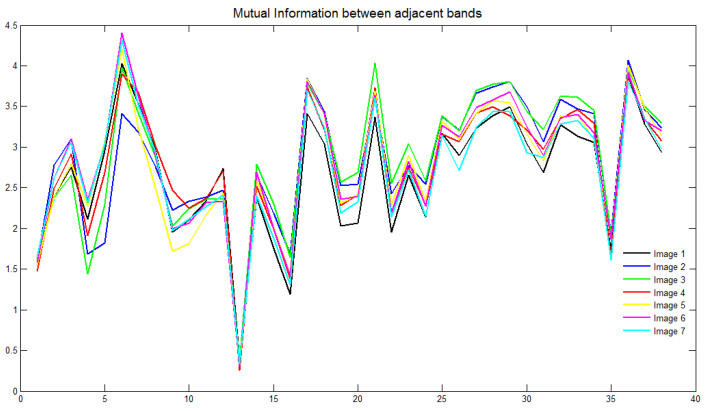
Mutual information between thirty-nine bands adjacent: the plot is given here for seven random images from the database.

H(A|B) is the amount of uncertainty left in *A* in the case where *B* is known. MI(A,B) shows the reduction in the uncertainty of *A* by the knowledge of another random variable *B*, as per Equation (3). This value can otherwise be depicted as the amount of information that B contains regarding A, which can represent the similarity between them. The redundancy in the image bands can be captured with the calculation of mutual information between the neighboring bands. Higher values of MI come with the bands that are highly similar, and it is expected to have more similarity between the adjacent bands. Therefore, the redundancy can be traced out by a close investigation of the nearby bands. Figure 3 shows a plot of mutual information against the respective bands for several sample images from the database. It is evident that all the curves exhibit a similar pattern, suggesting that the bands to be retained for a dataset can be generalized based on this observation. This process requires the band-selection algorithm to be performed as an offline procedure, eliminating the need for repetition with new images collected from the same setup. Unlike existing methods, this approach aims to reduce computational time, achieving the desired efficiency. Mutual information (MI) is calculated between neighboring bands, and bands with an MI value below a specified threshold are retained. If multiple neighboring bands have similar MI values (above the threshold), the band with the highest information, determined by its entropy value, is selected. The band with the highest entropy, indicating maximum information, is kept. As the threshold is adjusted from high to low, the number of selected bands decreases. The whole band-selection procedure is applied to a random set of images from the dataset. We take approximately 25 images, with each belonging to the different class, and the resultant bands are noted. As inferred from Figure 3, the obtained set of bands is common for a majority of samples with a few exceptions. A majority-based rule could finally select the bands under each threshold condition.

### 2.3. Feature Extraction

The four textural features local binary pattern (LBP), uniform rotation-invariant LBP, local phase quantization (LPQ), and rotation-invariant LPQ are used in this paper for the experimentation. The LBP is a special case of a texture spectrum model [30], proposed by Ojala et al. Initially, the image is partitioned into blocks and subsequently into cells. For each pixel, the intensity difference with its neighboring pixels is captured as a binary code word. These binary values are then converted to decimal values, and a histogram is computed for each cell. This process results in a feature vector. By concatenating the feature vectors from all cells, the LBP feature descriptor is obtained. Various LBP variants exist, such as the uniform LBP and the uniform rotation-invariant LBP [31]. All of these variants will reduce the original LBP feature size by a representation that involves the same bin for several similar patterns.

The LPQ feature was proposed by Ville Ojansivu [32]. This technique is based on the quantized phase of the discrete Fourier transform (DFT), calculated over local image windows. The LPQ identifies texture by performing local computations at each pixel and concatenating the resulting codes into a histogram, similar to the local binary pattern (LBP) texture algorithm. This method has been applied in areas such as blurred face recognition and fingerprint liveness detection. The LPQ operator’s codes are robust against centrally symmetrical blur, and using only phase information makes it invariant to uniform illumination changes. Additionally, a rotation-invariant extension of the local phase quantization texture descriptor has been proposed [33]. This extension commences with the estimation of local characteristic orientation followed by the extraction of a binary descriptor vector. In both stages, the phase of the locally computed Fourier transform coefficients is used.

### 2.4. Classification

Once the feature extraction is performed from the images, a classifier needs to be applied to differentiate the various cell types. We are using the supervised learning technique support vector machine (SVM). The SVM is a discriminative classifier that is defined by a separating hyperplane. Given a labeled training data input, the algorithm outputs an optimal hyperplane that categorizes new data, that is the test dataset [34]. The separating hyperplanes can differ in the length of the separation margin that they introduce between the classes. Accordingly, the generalization error can also vary. The optimal margin hyperplane is computed in the feature space instead of the input space utilizing the kernel trick method. The SVM classifier was applied using the MATLAB software R2019b with the libsvm toolbox [35]. This classifier is built on a radial basis function kernel with the parameter estimations (cost ‘c’ and gamma ‘g’) using a grid search method. The classification algorithm utilizes 70% of the data for training and 30% for testing.

### 2.5. Performance

We have utilized accuracy as the metric to assess and compare the classification performance of the two image modalities (multispectral and RGB), as well as to evaluate the performance of the proposed band-selection method. Accuracy is defined as the percentage of correctly predicted instances from the total number of instances in the dataset. This metric offers a clear measure of how well the model classifies samples correctly.

## 3. Results

In this section, we present the experimental results, comparing the performance of multispectral and RGB imaging in automated tumor grading. We start with a detailed presentation of the classification accuracy achieved by each imaging modality, followed by a discussion of the improvements observed with our proposed band-selection algorithm. Additionally, we compare the computational complexity of the multispectral and RGB imaging techniques before presenting the results of the band-selection process.

### 3.1. RGB vs. Multispectral Imaging

Each of the feature extraction techniques were applied on the multispectral image dataset, and the results were already presented in [36]. Similar methods were applied over the RGB images of the four classes of pathological samples. The images were split in the same way as the multispectral ones in order to enlarge the database. All the classification accuracies were based on the 50-fold data shuffling method of holdout validation [37]. The hyper parameters (cost ‘c’ and gamma ‘g’) determined for the SVM classifier using the grid search method are listed in Table 1. The RBF kernel was used in all instances.

**Figure 4:**
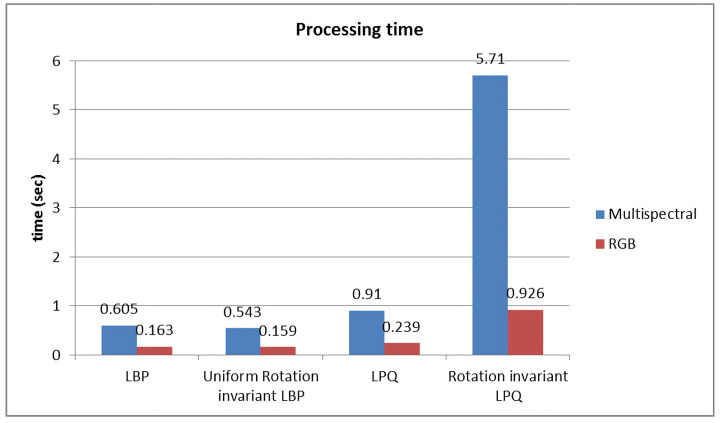
Computational time for the algorithms.

Table 2 shows that the multispectral imaging approach yields the best results across all methods, with the most significant accuracy improvement observed using the uniform rotation-invariant LBP (Uniform rLBP). This higher accuracy, however, comes at the cost of increased computational complexity. Figure 4 illustrates the processing time for each algorithm. The rotation-invariant LPQ (rLPQ) with multispectral imaging, which achieves the highest accuracy, requires considerably more time compared to its RGB counterpart. Therefore, selecting the relevant bands from the multispectral data is the optimal solution for this scenario.

### 3.2. Band-Selection Approach

We have implemented the proposed band-selection algorithm for tumor grading using the rotation-invariant LPQ features, as it is the most accurate method among the four listed in Table 2. The default local window size for the precomputed LPQ filters with the rotation-invariant LPQ descriptors was set to 9. We now test window sizes of 3, 5, 7, and 9 to investigate the corresponding band-selection results. Table 3 displays the results using all 39 bands. To determine the acceptable MI, we have selected four different thresholds, allowing for the selection of varying numbers of bands. Table 4 presents the classification accuracies with different numbers of bands and filter size variations.

A brief examination of the two tables indicates that the classification accuracies either improve or remain largely consistent compared to the results obtained using the full set of 39 bands. Specifically, accuracy increases from 87.70% to 87.91% when using only 19 bands. This suggests that eliminating redundant bands through band selection can notably reduce processing time. Moreover, focusing on features from relevant bands enhances accuracy. Even with just 10 bands, an accuracy of 87.15% is achieved, which is comparable to the result obtained without band selection.

To assess the significance of the band-selection algorithm, we conducted a similar analysis on another dataset. This dataset is a colorectal tumor dataset [38] consisting of 29 images from three classes (dataset II). The database is enlarged in a similar manner, which generated 464 images containing 16 spectral bands each. Table 5 presents the classification accuracies achieved using the original set of 16 bands. The results are shown for various window sizes of 3, 5, 7, and 9 for the LPQ filters. The band-selection algorithm yields the results depicted in Table 6.

Similar to the previous dataset, the classification accuracy in this study improves with band selection. The initial accuracy of 92.21% using 16 bands has increased to 94.09% after removing six bands. This result underscores the benefits of band selection for enhancing classification accuracy, in addition to reducing computational complexity.

## 4. Discussion

Hyperspectral imaging (HSI) and multispectral imaging (MSI) have proven to be valuable for identifying various diseases and tissues, with cancer detection being the most common application. However, determining the optimal spectral range remains challenging [27]. Several studies have shown that MSI can outperform standard RGB imaging for cancer cell classification. For instance, a comparative performance study in [39] on breast tissue microarrays demonstrated that MSI consistently yielded better classification results than standard RGB images. Another study [40] found that MSI significantly improved automated analysis of single-stained bright-field images of breast tissue microarrays. The analysis of H&E-stained MSI images was shown to be superior to conventional RGB images in predicting colorectal cancer prognosis [41]. All these studies used MSI images in the visible range (420 to 720 nm). Conversely, Ref. [42] used MSI in the near-infrared (VNIR) spectral range (400–1000 nm) to differentiate between normal and tumor breast cancer cells, comparing the results to synthetically generated RGB images. However, the dataset in this study was very small, consisting of only two patients. In contrast, our study compared the classification performance of multispectral and RGB imaging using a more comprehensive dataset (164 slides, 151 patients, four types of colorectal tissues, 1150 nm to 1650 nm NIR wavelength bands, in addition to visible bands). Our findings indicate that MSI outperformed RGB imaging when experimented with four different textural features.

Regarding multispectral and hyperspectral imaging techniques, we have to trade-off their benefits with the computational complexity due to increased dimensionality. Selecting the relevant bands from the numerous ones is a critical task, and we should ensure that the significant bands are still preserved after band selection. The band-selection algorithm in [43] utilizes the mutual information concept, where the mutual information is calculated between each band and the reference map. This map refers to the ground truth map in which each pixel is correctly assigned to a class. A higher MI indicates more resemblance to the ground truth, and those specific bands will be selected. Reference [44] is also based on a similar idea, with the reference map is generated using a priori knowledge of the scene. Redundant neighboring bands, having small differences in the MI values, are further excluded from the selection. The mutual information calculation in [45] is based on spatial-entropy-based mutual information (SEMI) and relies on the reference ground truth image. A higher SEMI indicates that the bands are to be selected. Band distance allows one to take distant bands to avoid correlation between adjacent bands. Maximum discrimination and information (MDI) based on the joint MI is defined in [46] using both labeled and unlabeled samples, which make up a semi-supervised criterion for band selection. To maximize the discriminative information of the selected bands, the DIR approximation method selects the bands having maximum relevance with the class label with the help of labeled samples. All of the methods described to date require a reference ground truth map for the computations. Moreover, the proposed approaches will be applicable only for such datasets as AVIRIS 92AV3C, as described in those works. The methodology in [47] is different in the sense that it does not need a ground truth map. This method follows a series of procedures, such as dissimilarity measurements between each set of bands based on mutual information and the Kullback–Leibler divergence method, a hierarchical clustering to group the bands based on the dissimilarity matrix for the entire band set, and finally, the identification of one representative from each cluster. The application of this band selection for each sample prior to the feature extraction and classification stages adds an increased computational burden, as previously mentioned.

Compared to other methods, our band selection is a simpler approach and computationally less complex. Since the selection of bands is performed as an offline task, the main algorithms will run normally with reduced time due to the reduced number of bands. The algorithms mentioned in [47] itself consume a major amount of time, which may hinder the objective of shortening the processing time. Our experiments with dataset I have shown that, with the 19 bands (half the number of original bands), the accuracy could be improved. Except with the filter window size of three, all other classification accuracies have increased. With the reduction in the number of bands, accuracies seem to increase first, reach a maximum, and then, tend to decrease. Even with 10 bands, the results did not deteriorate notably. This finding demonstrates that our band-selection algorithm could extract the most beneficial bands for the classification. The results on dataset II indicate major improvements for the classification accuracy over [36,38], which are based on similar feature extraction methodologies. Extracting features from just 5 out of the total of 16 bands can yield higher accuracy than using the entire set of bands. This highlights our algorithm’s capability to eliminate redundant bands using mutual information and identify prominent bands based on image entropies.

## 5. Limitations and Future Work

Despite the promising results, our study has a few limitations. Our study focused on colorectal tumors, which may limit the generalizability of our findings to other types of cancers or diseases. Another limitation is the potential variability in imaging conditions and sample preparations, which could affect the reproducibility of our results across different datasets and settings.

We plan to expand our study to include a wider variety of tissue types and other forms of cancer to test the generalizability of our findings. Standardizing imaging protocols and sample-preparation methods will be crucial to enhancing the reproducibility of our results. Additionally, we will investigate the integration of advanced machine learning algorithms with multispectral imaging data to develop more sophisticated and accurate classification models.

## 6. Conclusions

We have performed a study to demonstrate the advantages of multispectral imaging over RGB imaging in the aspect of automated tumor grading. The results on a four-class colorectal tumor dataset indicate an improvement of classification accuracy from 80.71% for RGB to 86.05% with the multispectral image data. An increase in data dimensionality will be clearly accompanied by a rise in computational complexity. This finding makes sense of band-selection approaches to remove the redundant bands. Our proposed algorithm for band selection identifies the similarity between neighboring bands and selects the relevant ones. The offline band-selection procedure can identify the set of bands that should be retained, which will be the same for all images captured from a common setup. This finding ensures that the computational complexity declines in further experiments. Moreover, the classification accuracy increases when processing with the selected bands alone, even if it is less than half the original number of bands. Experiments on two colorectal tumor datasets have supported the hypothesis. In our multispectral image dataset, the accuracy improved from 86.05% to 87.91% with the 19 bands selected from the whole 39-band multispectral dataset. The results on the other dataset showed that the band selection yielded accuracy values of 92.21% and 94.09% using 16 whole bands and 10 selected bands, respectively. Compared to previous methods [36,38], our method outperformed those by 2.6% and 2.8%, respectively, for dataset II.

## Figures and Tables

**Table 1:** Estimated hyper parameters for SVM classifier.

Method	(c, g) for Multispectral Images	(c, g) for RGB Images
LBP	8, 0.0625	8, 2
Uniform rLBP	4, 1	8, 2
LPQ	2, 0.0625	8, 0.0625
rLPQ	8, 0.0625	8, 0.25

**Table 2:** Comparison of classification accuracies with multispectral and RGB images.

Method	Multispectral Images (320 × 256 × 39)	RGB Images (320 × 256 × 3)
LBP	77.86	65.32
Uniform rLBP	83.61	66.99
LPQ	67.52	65.29
rLPQ	86.05	80.71

**Table 3:** Classification accuracies without band selection (for filter window sizes of 3, 5, 7, and 9).

Filter Size	3	5	7	9
Rotation-Invariant LPQ	86.11	86.39	87.70	86.05

**Table 4:** Classification accuracies with band selection.

No. of Bands ^1^	22	19	17	10
Filter Size				
3	86.17	85.30	83.62	78.86
5	86.83	87.31	86.82	85.12
7	87.87	87.91	87.50	87.15
9	86.52	86.77	86.19	85.37

^1^ An MI threshold of 3.25 leads to the selection of 22 image bands from the 39-dimensional multispectral image cube and similarly for 19 bands (MI < 3), 17 bands (MI < 2.75), and 10 bands (MI < 2.5).

**Table 5:** Classification accuracies without band selection for dataset II.

Filter Size	3	5	7	9
Rotation-Invariant LPQ	92.21	91.96	91.68	90.32

**Table 6:** Classification accuracies with band selection for dataset II.

No. of Bands ^1^	22	19	17	10
Filter Size				
3	92.57	93.83	93.77	90.11
5	91.44	94.09	93.96	92.75
7	91.22	92.24	92.60	92.86
9	90.73	92.39	92.55	91.84

^1^ An MI threshold of 3 leads to the selection of 13 image bands from the 16-dimensional multispectral image cube and similarly for 13 bands (MI < 3), 10 bands (MI < 2.75), 8 bands (MI < 2.5), and 5 bands (MI < 2.25).

## Data Availability

The data presented in this study are available upon request from the corresponding author.
